# Grand Challenge: Understanding Survival Paradoxes in Epidemiology

**DOI:** 10.3389/fpubh.2013.00003

**Published:** 2013-04-02

**Authors:** Jimmy T. Efird, Wesley T. O’Neal, Whitney L. Kennedy, Alan P. Kypson

**Affiliations:** ^1^Department of Cardiovascular Sciences, East Carolina Heart Institute, Brody School of Medicine, East Carolina UniversityGreenville, NC, USA; ^2^Center for Health Disparities Research, Brody School of Medicine, East Carolina UniversityGreenville, NC, USA

## Introduction

Survival paradoxes are commonly observed in the literature. This phenomenon describes the association of certain risk factors with negative outcomes in the general population and the opposite effect in certain subpopulations and vice versa. Overall, the field is poorly understood. The counter-intuitive findings reported in the literature have contributed to confusion among clinicians regarding the appropriate treatment of conventional risk factors in patients with chronic diseases. Below we review two such paradoxes in kidney and cardiovascular disease epidemiology and discuss possible explanations of these findings.

## Survival Advantage among Black Hemodialysis Patients

Despite racial disparities among blacks in socioeconomic position, education, lifestyle factors, comorbid conditions, access to medical care, and utilization of health services, a reverse survival advantage is seen in hemodialysis patients. For example, conventional cardiovascular risk factors such as black race, hypercholesterolemia, hypertension, and obesity are associated with increased survival among hemodialysis patients (1).

One of the first comprehensive studies to report a survival paradox among hemodialysis patients was conducted in eastern Michigan among 594 diabetic end-stage renal disease (ESRD) patients (2). Risk of death was nearly 45% [Hazard Ratio (HR) = 0.55, 95% Confidence Interval (CI) = 0.44–0.69] lower in black hemodialysis patients compared with white hemodialysis patients after adjusting for factors related to survival in their database (type of diabetes, comorbid conditions, and demographic factors) (2). This study was consistent with the point prevalence results from the U.S. Renal Data System report showing a lower annual adjusted (age, gender, race, primary diagnosis, and vintage) death rate (per 1000 patient years) of 187 for black hemodialysis patients compared with 223 for white hemodialysis patients (3). Another analysis of the U.S. Renal Data System database reported that the survival paradox persisted after adjustment for case-mixed differences [Relative Risk (RR) = 0.78, 95% CI = 0.71–0.86], transplantation rates (RR = 0.83, 95% CI = 0.75–0.91), withdrawal from dialysis (RR = 0.81, 95% CI = 0.73–0.90), and initial treatment mortality (RR = 0.79, 95% CI = 0.71–0.87) (4).

The survival advantage observed in the above studies, if not artifactual, may be due to differences in genetics, nutritional status, inflammation, and sensitivity to dialysis (5, 6). However, results of the American arm of the first phase of the Dialysis Outcomes and Practice Patterns Study, a prospective observational study of 6677 patients between 1996 and 2001, found that the cumulative adjustment for laboratory (bicarbonate, calcium-phosphorus product, ferritin, hemoglobin, potassium, transferrin saturation, and white blood cell count) and hemodialysis (treatment time, systolic blood pressure pre-dialysis, and ultrafiltration volume) measures, in a model that already adjusted for conventional cardiovascular disease risk factors, resulted in a near null HR for race as a predictor of survival among hemodialysis patients (HR = 0.97, 95% CI = 0.85–1.11) (7). Although the effect size was diminished in the latter study, it is unclear whether over-adjustment by factors in the causal pathway explains the result.

## Obesity Survival Advantage in Cardiovascular Disease

Recently, several longitudinal studies have shown that obesity is associated with improved survival compared with normal weight individuals. Data from the PREMIER and TRIUMPH national registries of patients hospitalized with acute myocardial infarction observed a decreased risk of mortality at 1 year among patients with body mass index (BMI) ≥ 35 kg/m^2^ compared with normal weight individuals (HR = 0.59, 95% CI = 0.37–0.91) (8). Patients from the APPROACH registry that received coronary artery bypass grafting (CABG) for the treatment of coronary artery disease had a lower risk of mortality if their BMI ranged from 30.0 to 34.9 kg/m^2^ compared with normal weight patients (HR = 0.75, 95% CI = 0.61–0.94) (9). Decreased HRs also were noted for BMI categories 25.0–29.9 kg/m^2^ (HR = 0.85), 35.0–39.9 kg/m^2^ (HR = 0.89), and >40.0 kg/m^2^ (0.77), although upper CIs spanned unity.

The so-called obesity paradox also has been reported in a national examination of 348,341 isolated CABG patients from the Society of Thoracic Surgeons Adult Cardiac Surgery Database demonstrating that high (BMI > 25 kg/m^2^) across postoperative time periods (30 days to > 2 years) was significantly associated with decreased mortality compared with normal weight individuals (*p* < 0.05) (10). However, the decreased effect sizes were nominal ranging from 0.79 to 0.94.

While the explanation for the obesity paradox among patients with cardiovascular disease is unknown, the results possibly may be attributable to having better nutritional reserves to protect against mortality. Alternatively, treatment and referral biases could account for these differences. Physicians may be more likely to refer obese patients for treatment since they are perceived to be a high-risk group for developing coronary artery disease.

## Grand Challenge

Reverse survival paradoxes may reflect the observational nature of epidemiologic studies. While such studies are excellent for the generation of hypotheses, they are unable to prove causality. Whether the reverse epidemiologic effects are real or merely reflect the consequences of other underlying factors (e.g., residual confounding, violation of the independent censoring assumption, inappropriate adjustment, differential withdrawal from study participation, or Simpson’s paradox) remains controversial, especially given the lack of convincing underlying pathophysiological evidence. Explaining survival paradoxes in the field of epidemiology remains a grand challenge for future researchers in this field.
